# Estimation of the molecular weight of nanoparticles using a single small-angle X-ray scattering measurement on a relative scale

**DOI:** 10.1038/s41598-021-87133-x

**Published:** 2021-04-08

**Authors:** Alexander Zhigunov, Josef Pleštil

**Affiliations:** Institute of Macromolecular Chemistry, Czech Academy of Sciences, Heyrovského nám. 2, 162 06 Prague 6, Czech Republic

**Keywords:** Computational methods, Polymers, Characterization and analytical techniques

## Abstract

Both small-angle scattering methods, X-rays (SAXS) and neutrons (SANS) rank among the methods that facilitate the determination of the molar mass of nanoparticles. Using this measure, aggregation or degradation processes are easy to follow. Mono- and multichain assemblies of nanoparticles in solution could be resolved, swelling ratio can also be obtained. In this work, we present a method that allows extraction of additional information, including molecular weight, from a single scattering curve, even on a relative scale. The underlying theory and step-by-step procedure are described.

## Introduction

Small-angle scattering is a well-established technique to obtain information about the thermodynamic state of inhomogeneities in the scale ranging from 10 Å up to 1000 Å. One of the parameters that is usually obtained during data processing is the molecular weight of moieties. Both small-angle scattering methods, X-rays (SAXS) and neutrons (SANS) rank among the methods that facilitate the determination of the molar mass of nanoparticles. Using the molecular weight, aggregation or degradation processes are easy to follow. Mono- and multichain assemblies of nanoparticles in solution and estimates of the swelling ratio can also be obtained, among other applications.

A particular application of this parameter is its use as complementary information in protein X-ray crystallography. Protein crystals may contain nonphysiological protein–protein interfaces that are stabilized by crystal packing^1^. As soon as the degree of oligomerization is able to be determined using the molecular weight obtained from SAXS, it could be a good tool to choose biologically relevant complexes in a crystal. Different approaches are used to extract this useful information from scattering curves.

The classical (conventional) procedure is based on the relation

1 $${\text{M}}_{{\text{w}}} = \frac{1}{{\text{c}}} \cdot \frac{{{\text{N}}_{{\text{A}}} }}{{\Delta {\text{b}}^{2} }} \cdot \frac{{{{d\Sigma }}}}{{{{d\Omega }}}}\left( 0 \right)$$

where $\frac{d\Sigma }{d\Omega }(0)$ is the differential cross-section of coherent scattering extrapolated to a zero scattering angle, *c* is the particle concentration, *N*_*A*_ is the Avogadro number,

2 $$\Delta b=b-\stackrel{-}{V}{\rho }_{0}$$

is the excess scattering amplitude, *b* and $\overline{V}$ are the scattering amplitude and partial volume of the solute, respectively, and $\rho_{0}$ is the scattering density of the solvent. The calculation of *M*_*w*_ from Eq. (1) requires scattering intensities on an absolute scale and the knowledge of the contrast factor *Δb*. This information is not always available. The theoretical calculation of the contrast factor requires knowledge of a partial specific volume with high accuracy. The accuracy of the molecular weight determined using this approach was estimated to be approximately 10% on average^2^, but reached 40% deviations for some cases.

The mean-square fluctuation of scattering density is calculated using the following equation reported by Kratky^3^:

3 $$\frac{\overline{{(\Delta \rho )}^{2}}}{c} =\frac{1}{2 {\pi }^{2}}{\int }_{0}^{\infty }\frac{1}{c}\cdot \frac{d\Sigma }{d\Omega }\left(q\right)\cdot {q}^{2}\cdot dq$$

Here are the equations for the mean-square fluctuations of the scattering density, volume fraction, *ν*, and scattering density of the solute, *ρ*_*1*_:

4 $$\overline{({\Delta \rho )}^{2}}=\nu (1-\nu ){({\rho }_{1}-{\rho }_{0})}^{2}$$

5 $$\nu =c\overline{V}$$

6 $${\rho }_{1}= \frac{b}{\overline{V}}$$

Using Eqs. (2), (5) and (6), Eq. (4) is rewritten in the following form:

7 $$\overline{{(\Delta \rho )}^{2}}= c\cdot \left(\frac{{\Delta b}^{2}}{\overline{V}}-c\cdot {\Delta b}^{2}\right)$$

Interestingly, $\frac{\overline{{(\Delta \rho )}^{2}}}{c}$ represents the linear equation of the concentration *c*. This equation also shows the dependence of the scattering amplitude on the concentration:

8 $${\Delta b}^{2}= \frac{\overline{{(\Delta \rho )}^{2}}}{c}\cdot \left(\frac{\overline{V}}{1-c\overline{V}}\right)= \frac{\overline{{(\Delta \rho )}^{2}}}{c}\cdot \left(\frac{\overline{V}}{1-\nu }\right)$$

Substituting *Δb*^*2*^ from Eq. (8) and returning to classical equation of molecular weight determination, we obtain the following equation:

9 $${M}_{w}= \frac{1}{c}\cdot {N}_{A}\cdot \frac{\frac{d\Sigma }{d\Omega }\left(0\right)}{\frac{\overline{{\left(\Delta \rho \right)}^{2}}}{c}\cdot \left(\frac{\overline{V}}{1-\nu }\right)}= {N}_{A}\cdot \frac{\left(1-\nu \right)\cdot \frac{d\Sigma }{d\Omega }\left(0\right)}{\overline{{\left(\Delta \rho \right)}^{2}}\cdot \overline{V}}={N}_{A}\cdot \frac{c\cdot \left(1-\nu \right)\cdot \frac{d\Sigma }{d\Omega }\left(0\right)}{\overline{{\left(\Delta \rho \right)}^{2}}\cdot \nu }={N}_{A}\cdot \frac{c\cdot \frac{d\Sigma }{d\Omega }\left(0\right)}{{({\rho }_{1}-{\rho }_{0})}^{2}\cdot {\nu }^{2}}$$

In 1988, Pleštil identified a relation between the scattering intensity and the contrast factor^4^, which enables the molar mass to be determined without absolute intensities and the contrast. At least two SAXS curves for different concentrations are necessary. The use of additional scattering curves increases the accuracy of the contrast factor determination. Advantages and limitations of this approach are described in the study by Pleštil et al.^5^.

Oliveira Neto et al.^6^ proposed a novel method to determine the molecular weight of proteins in dilute solutions using the experimental data from a single SAXS curve measured on a relative scale. The proposed procedure could be applied to monodisperse systems either in a monomeric or multimeric state. A number of prerequisites exists in this method, namely, the monodispersity of the sufficiently diluted system, the homogeneity of the proteins under investigation and a statistically isotropic solution. Unlike the procedure reported by Oliveira, which is appropriate for diluted systems, our procedure works for concentrated systems.

According to Rambo and Tainer^7^, only limited structural parameters are able to be derived from SAS curves in some cases. Their approach allows researchers to obtain structural information about non-compact or folded particles independent of the concentration.

A thorough review was recently published by Korasick and Tanner, in which they have summarized the computational methods that are used to determine the molecular mass of a protein^8^. They proposed to divide *M*_*w*_ determination methods into three categories: (1) empirical methods, (2) $\frac{d\Sigma }{d\Omega }(0)$-based methods and (3) invariant-based methods. In our approach, we use the invariant-based method, but with the requirement of a known concentration.

Although this paper is not covering data collection and primary processing, SAXS curves are presumed to be available for interpretation, namely, high-quality data have been collected and the background has been carefully subtracted.

## New approach in the determination of the molecular weight from a single scattering curve with an arbitrary scale

### Case of a high concentration

The novel method proposed here can be applied to determine the molar mass of nanoparticles using experimental data collected on a relative scale for only one concentration. The method is based on the previous approach described by Plestil^9^, in which he determined the excess scattering amplitude from small-angle scattering data. Notably, *Δb* was calculated using a plot of the concentration dependence versus integrated intensities:

10 $$\Delta {b}^{2}=-\frac{dZ}{dc}$$

11 $$Z=\frac{1}{2{\pi }^{2}}\underset{0}{\overset{{q}_{m}}{\int }}\frac{1}{c}\frac{d\Sigma }{d\Omega }\left(q\right){q}^{2}dq$$

**Figure 1:**
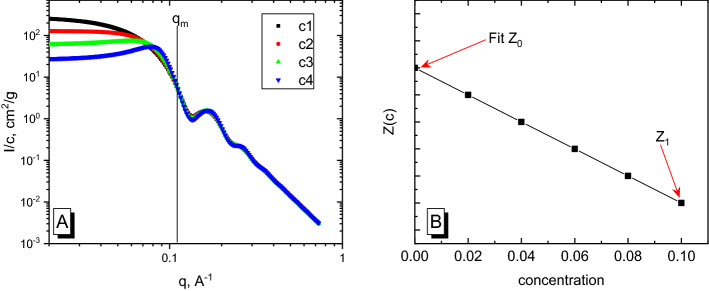
**(A)** Plot of the scattering intensity divided by the concentration versus the scattering vector. The behavior of scattering curves is independent of the concentration for *q* > *q*_*m*_. **(B)** Plot of the integrated (*I·q*^*2*^) versus concentration. Arrows are pointing to the data points that will be available when proposed method is applied.

The upper integration limit *q*_*m*_ was chosen to ensure that at *q* > *q*_*m*_, the normalized scattering intensity $\frac{1}{c}\cdot \frac{d\Sigma }{d\Omega }\left(q\right)$ is independent of the concentration (see Fig. 1A). The reason to determine *q*_*m*_ is that intensities at a higher *q*-range are often noisy simply because of a poor statistic or are affected by the background.

Here, we are providing a solution for the case when only one scattering curve is available (single concentration), the intensity scale is unknown, but the concentration is known (or could be measured or calculated from the linear absorption coefficient). The common approach is similar to the method discussed by Plestil, but when only one experimental curve is available, the plot of concentration dependence versus integrated intensity only contains one point (Fig. 1B; the point is labeled as Z_1_). When scattering curves for different concentrations are available, all points should lie on a straight line, according to Eq. (7). Those points are not labeled in Fig. 1B. The only crucial factor is the knowledge of the exact concentration. The Z_i_ value decrease with concentration (Fig. 1B), which mean that the downward trend of scattering intensity at low-*q* range is a prerequisite. This usually indicates repulsive inter-particle interactions.

The described procedure could also be used as a criterion for assessing the correctness of the form factor, while comparing obtained values with predicted. A different approach to solving this problem was proposed by D. Franke, C. Jeffries, and D. Svergun^10^.

Because all scattering curves at *q* > *q*_*m*_ have the same profiles (after normalization to the concentration), we are able to fit the scattering curve for this independent range of scattering vectors. The structure factor should not be involved. If the chosen fitting function is correct, we will obtain a theoretical scattering curve that corresponds to the zero concentration. Thus, we will obtain another point in the plot shown in Fig. 1B (the point is labeled as Fit Z_0_). According to Eq. (10), the slope of the line between these two points corresponds to *Δb*^*2*^ if the experimental scattering curve was on absolute scale. The equation for molecular weight determination takes form:

12 $${M}_{w}={N}_{A}\frac{{I}_{fit}(0)}{\frac{1}{2{\pi }^{2}}\left({\int }_{0}^{{q}_{m}}\frac{1}{c}{I}_{fit}(q){q}^{2}dq-{\int }_{0}^{{q}_{m}}\frac{1}{c}{I}_{exp}(q){q}^{2}dq\right)}$$

If the intensity is not on the absolute scale and should be multiplied by some scale factor, the same scale factor will be included in the numerator and denominator. Therefore, this relation is useful for determining the molecular weight without absolute intensities and with only a single experimental scattering curve. The proposed method will provide some benefits and limitations that will be discussed below.

### Case of a low concentration

In the case of a low concentration, we used the equations described above. Equations (3) and (8) lead to the equation for calculating *Δb*^*2*^ and *M*_*w*_:

13 $$\Delta {b}^{2}=\frac{\frac{1}{2{\pi }^{2}}\underset{0}{\overset{\infty }{\int }}I{q}^{2}dq}{c\left(\frac{1}{\stackrel{-}{V}}-c\right)}$$

14 $${M}_{w}=\frac{{I}_{0}}{c}{N}_{a}\frac{c\left(\frac{1}{\stackrel{-}{V}}-c\right)}{\frac{1}{2{\pi }^{2}}\underset{0}{\overset{\infty }{\int }}I{q}^{2}dq}=\frac{2{\pi }^{2}{I}_{0}{N}_{a}\left(\frac{1}{\stackrel{-}{V}}-c\right)}{\underset{0}{\overset{\infty }{\int }}I{q}^{2}dq}$$

Or using the volume fraction (Eq. 5):

15 $${M}_{w}=\frac{2{\pi }^{2}{I}_{0}{N}_{a}c\left(\frac{1}{\upsilon }-1\right)}{\underset{0}{\overset{\infty }{\int }}I{q}^{2}dq}$$

The proposed above equation for molecular weight estimation (Eq. 15) is applicable for the smooth data only. This requirement is hardly achievable in a single measurement, but could be obtained by additional experiments with higher concentrations. There are several criteria for equation choice. One should try this M_w_ estimation if (1) the obtained scattering curve is smooth; (2) I(0) could be estimated (on a relative scale); (3) the influence of the structure factor on the scattering curve is negligible. As this method is based on the same idea of invariant usage, but provide additional requirements, further on in examples we will stick to high-concentration cases.

## Simulations of particles with a Percus–Yevick interaction

We have confirmed the validity of suggested method using several datasets, including simulated data and measured data. For simplicity, we have started with a simulated dataset. We have calculated theoretical scattering curves that correspond to several polymers dissolved in different solvents. We assume that we were obtaining spherical particles with a radius of 90 Å in all simulated combinations. The hard sphere structure factor with Percus–Yevick closure with a volume fraction of 0.2 was used to describe the interactions. The structure factor must be included, as we are modeling a non-zero concentration behavior. The polydispersity of the particle size was set to the Gaussian type with the standard deviation exceeding the mean value of 0.05. We were using SasView V4.1.2 software to simulate scattering curves^11^.

Parameters of different combinations are described in Table 1. The same parameters were used for each of the simulations, excluding the scattering length densities. Thus, volume is the same for all:

16 $$V= \frac{4}{3}\pi {r}^{3}=3.054\cdot {10}^{6} {\AA }^{3}$$

**Table 1:** Overview of the parameters for the two-phase systems used in simulations.

Combination	Polymer density, g/cm^3^	Solvent density, g/cm^3^	Polymer SLD, ·10^–6^ Å^-2^	Solvent SLD, ·10^–6^ Å^-2^	Concentration, wt.%	Mw, g/mol	Δb^2^, cm^2^/g^2^
PS/H_2_O	1.04	1	9.5156	9.4691	0.208	1.91·10^6^	2.00·10^17^
PS/C_2_H_5_OH	1.04	0.791	9.5156	7.6022	0.208	1.91·10^6^	3.38·10^20^
PS/CHCl_3_	1.04	1.486	9.5156	12.4894	0.208	1.91·10^6^	8.18·10^20^
PMMA/H_2_O	1.17	1	10.749	9.4691	0.234	2.15·10^6^	1.20·10^20^
PP/H_2_O	0.855	1	8.2952	9.4691	0.164	1.57·10^6^	1.88·10^20^

Recalculation of the volume fraction into a concentration was performed using the following equation:

17 $$c= \upsilon *\rho$$

where *c* is the concentration, *υ* is the volume fraction and *ρ* is the density.

The theoretical molecular weight for the simulated spherical particles was calculated from the volume:

18 $${M}_{w}=V\cdot \rho \cdot {N}_{A}$$

From this point, let us assume that we know nothing about our system. We only have our scattering profiles and the concentrations used.

### Step-by-step procedure

We have used SASFit software^12^ to fit the scattering curve. The software provides several metrics to show the “goodness-of-fit”, while trying to minimize solely χ^2^. Details and criteria of acceptable values for each metric could be found in documentation to SASFit software^12^. The sphere function with the Schultz-Zimm size distribution was used. We have obtained following parameters: *R* = 90.71 Å, *σ* = 0.049, and scattering contrast, *Δη* = 1.176·10^–7^.

The intensity extrapolated to *q* = 0 was obtained from the fitting procedure or could simply be calculated using an equation valid for spherical particles:

19 $$I\left(q\to 0\right)={\left(\frac{4}{3}\pi {R}^{3}\Delta \eta \right)}^{2}=0.1352\space {\mathrm{cm}}^{-1}$$

**Figure 2:**
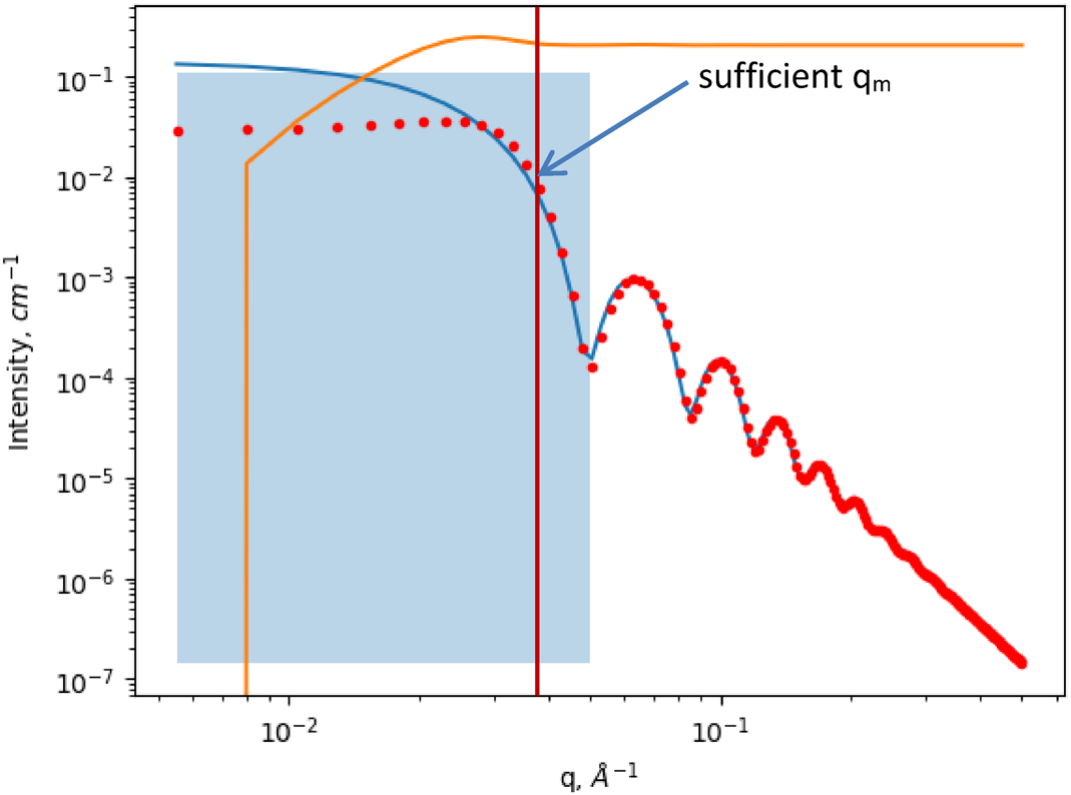
Simulated scattering curve of polystyrene in water (red dots); best fit (blue line); invariant calculation range, with the right side equal to *q*_*m*_ (blue rectangle); cumulative invariant calculation (orange line) multiplied by the factor of 10^–18^ to make its scale closer to the scattering curves.

The orange line in Fig. 2 corresponds to the difference in cumulative invariants, which was calculated using Eq. (11). Cumulative integration was intentionally performed in the whole region (orange line in Fig. 2). First, *q*_*m*_ was chosen based on its behavior, namely, we chose any *q* that was larger than the point at which the value of the cumulative invariant becomes a constant. For this case, the *q*_*m*_ = 0.05 was chosen. On the other hand, the quality of the fit is easy to determine based on the behavior of cumulative invariant difference.

We calculated the contrast factor by dividing the difference in invariants by the concentration ($\frac{dZ}{dc}$) using Eqs. (10) and (11). Here, *dc* is simply equal to the concentration we modeled. We have obtained the value of ($-\frac{dZ}{dc}$) = *Δb*^*2*^ = 2.05·10^17^ cm^2^/g^2^, and then, using Eq. 12 we have calculated *M*_*w*_ as 1.90·10^6^ g/mol. Both values, *Δb*^*2*^ and *M*_*w*_, are very similar to the original theoretical values of the simulated model. A similar procedure was performed for other polymer/solvent pairs. The results are presented in Table 2.

**Table 2:** Parameters, obtained by fitting procedure and the method applied.

combination	Radius, Å	Δη, cm/g	Calc. I(0), cm^−1^	Calc. Δb^2^, cm^2^/g^2^	Calc. M_w_, g/mol	Theor. M_w_, g/mol
PS/H_2_O	90.71	1.18·10^–7^	0.130	2.06·10^17^	1.83·10^6^	1.91·10^6^
PS/H_2_O × 50	90.23	8.36·10^–7^	6.52	1.09·10^19^	1.84·10^6^	1.91·10^6^
PS/C_2_H_5_OH	90.56	4.85·10^–6^	219.34	3.53·10^20^	1.80·10^6^	1.91·10^6^
PS/CHCl_3_	90.51	7.54·10^–6^	530.12	8.52·10^20^	1.81·10^6^	1.91·10^6^
PMMA/H_2_O	90.50	3.25·10^–6^	98.49	1.25·10^20^	2.03·10^6^	2.15·10^6^
PP/H_2_O	90.1	2.99·10^–6^	83.36	2.08·10^20^	1.47·10^6^	1.57·10^6^

Coincidence with the theoretical values is very good, but not perfect. Insignificant differences are explained by the use of a real fitting procedure, which leads to minor deviations in the parameters. The fitted curve does not completely describe the behavior of “experimental points” at the lower *q*-region (lower than *q*_*m*_), and a perfect fit would result in a higher *I(0)* value, allowing the calculated *M*_*w*_ to coincide with the theoretical value. The example of the method to choose correct *q*_*m*_ value is shown as an arrow in Fig. 2. The behavior of the cumulative integral could be used as a criterion. The choice depends on the quality of the experimental data. A sufficient *q*_*m*_ should be chosen at the point where the difference in cumulative integrals (orange line) becomes a constant. Furthermore, the choice of *q*_*m*_ should fulfill two basic rules: features of the scattering curve must be included in fit procedure and the fitting region should not include lower *q*-values, which are influenced by interactions. This approach will provide us a wide range of *q*-values, where the result is not sensitive to the chosen *q*_*m*_. If the experimental data from several concentrations are available, the choice of *q*_*m*_ is rather straightforward: it is the point where curves from different concentrations coincide on the scale *I/c* (as depicted in Fig. 1A).

Notably, the value of *Δb*^*2*^ could be obtained directly from graph, if the scattering curve was on an absolute scale. In this case, the cumulative invariant difference should be divided by the concentration, and then a value in the region larger than *q*_*m*_ would be selected.

### Relative scale proof

We intentionally multiplied the simulated scattering curve for PS/H_2_O by the factor of 50 (labeled as “PS/H_2_O × 50” in Table 2) to show the independence of the molecular weight determination on an absolute scale. The value of the contrast is not correct in this case; nevertheless, we are still obtaining a correct value for the molecular weight.

Small deviations of the obtained parameters from the initial parameters might be explained by the intentionally involved polydispersity, relatively high volume fraction and limited number of “experimental” points. With these factors minimized, the obtained and initial parameters coincide.

Thus, the proposed method is correct and potentially useful for estimating *M*_*w*_ and *Δb*^*2*^ from real scattering curves.

### Verification of the chosen model

The method is very sensitive to the obtained *I(0)* value. Thus, in most cases, this parameter should not be determined from the Guinier approximation, particularly for samples with a high concentration. Therefore, the proposed approach is a good indicator for determining the correct model. We used simulated data for PS/H_2_O (first row from Table 1) and attempted to fit it to the spheroid function with *ν* = 1.05 to confirm this hypothesis.

**Figure 3:**
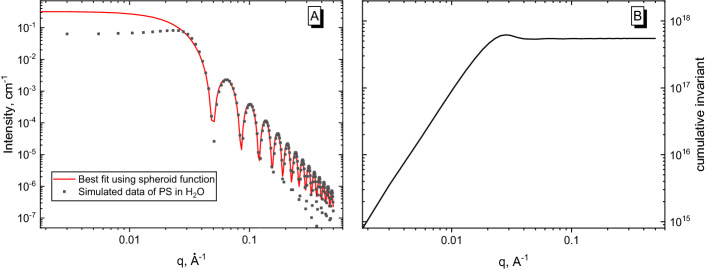
**(A)** Simulated scattering curve for the PS/H_2_O pair (black squares) and best fit using a spheroid function (red line); **(B)** cumulative invariant difference.

The fit shown in Fig. 3A resembles the actual data. By applying the proposed method, we obtained *Δb*^*2*^ = 5.46·10^17^ cm^2^/g^2^ and *M*_*w*_ = 1.71·10^6^ g/mol. If our data were on an absolute scale, each of these parameters could be used to judge the chosen model. Comparing these results with values from Table 1, reveal that *Δb*^*2*^ is the more sensitive indicator. Figure 3 (B) is the graphical representation of cumulative invariant, where the saturation value, at higher *q*, coincides with obtained *Δb*^*2*^.

### Effects of size and polydispersity

**Figure 4:**
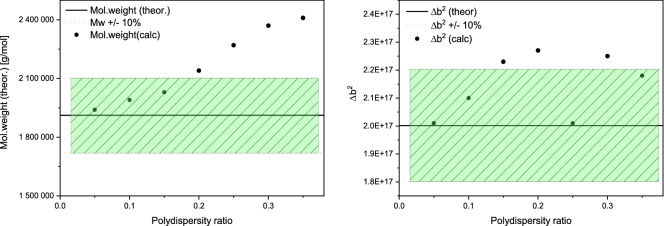
Acceptable accuracy window for the determination of *M*_*w*_ and *Δb*^*2*^.

Polydispersity is the one of the negative factors, but the method is still applicable within some reasonable PD ratios. We have simulated the polydispersity conditions for PS/H_2_O model to show the limits of the application. The results of the calculations are shown in Fig. 4, where the green window indicates an acceptable accuracy (within 10%) of the molecular weight determination. We have used an absolute intensity scale in the simulation and thus expected *Δb*^*2*^ to be in the acceptable range as well. Polydispersity drastically affects the behavior of scattering curve in the low *q*-region, where the accuracy of the method is very sensitive. Although we have obtained reasonable values for *Δb*^*2*^, the values of *M*_*w*_ deviated rather rapidly. We admit that samples with a polydispersity ratio greater than 0.2 would not produce an acceptable result, mainly because of an incorrect *I(0)* estimation.

### Three phase systems

We have conducted theoretical and practical examinations to determine what information can be extracted when core–shell system is analyzed.

For simplicity, we decided to simulate the behavior of a known poloxomer, Pluronic PE10500. In aqueous solution, it forms a hydrophobic core composed of polypropylene glycol (PPG) and shell of polyethylene glycol (PEG). According to the specifications, the percentage of PEG in the molecule is 50% and the molar mass is 3250 g/mol, with a bulk density = 1.03 g/cm^3^. Again, we have used SasView software to simulate scattering curves with the desired parameters, which are listed in Table 3:

**Table 3:** List of the parameters used to simulate scattering curve for Pluronic PE10500.

R (core) (Å)	Shell thickness (Å)	SLD (core) ·10^–6^ (Å^−2^)	SLD (shell) ·10^–6^ (Å^−2^)	SLD (H2O) ·10^–6^ (Å^−2^)	Polydispersity (core)	Polydispersity (shell)	Vol. fraction
60	16.5	8.0592	10.44	9.4691	0.1	0	0.15

**Figure 5:**
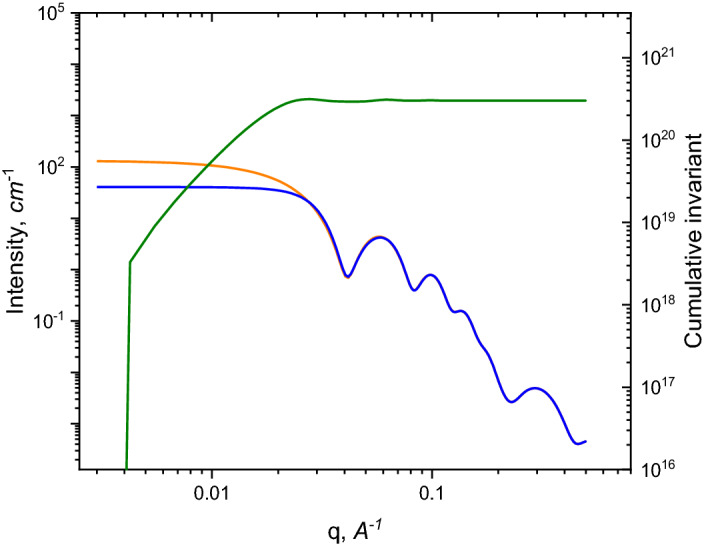
Simulated (blue) and fitted (orange) scattering curves for Pluronic PE10500. Cumulative invariant curve is drawn in green.

We have used our proposed procedure to calculate the molecular weight and *Δb*^*2*^. Resulting curves are depicted in Fig. 5. The obtained values are very promising: the calculation yielded an *M*_*w*_ = 1.15·10^6^ g/mol and *Δb*^*2*^ = 5.65·10^18^ cm^2^/g^2^. The calculation of the *M*_*w*_ from the volume of the particle using bulk density, results in *M*_*w*_ = 1.122 ·10^6^ g/mol. Unfortunately, we have not determined how to extract any meaningful information from the obtained *Δb*^*2*^ value.

## Real experimental data

In this section, we would like to show the application of the described model to several chosen experimental datasets.

### Poly(methyl methacrylate) in water

**Figure 6:**
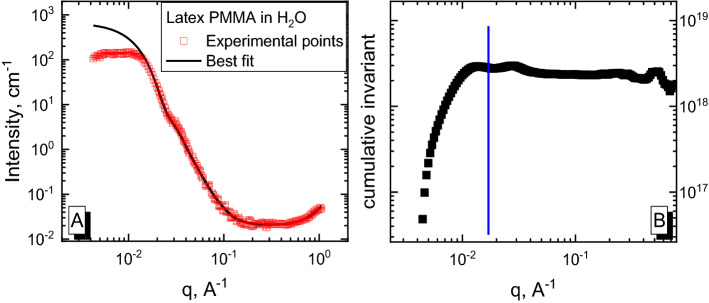
**(A)** Small-angle X-ray scattering curves of PMMA latex particles in water and its best fit; **(B)** appropriate cumulative invariant with the labeled *q*_*max*_ value as a vertical blue line.

Experimental data and fitting curves are presented in Fig. 6. In fact, we had a concentration series for this sample, namely, 9.26 wt.%, 4 wt.%, 0.92 wt.% and 0.46 wt.%. The curve in Fig. 6 corresponds to the highest concentration, 9.26 wt.%. We used the sphere model to describe the behavior of the particles. The size distribution is not very narrow, but a second maximum is still observed. Thus, a fitting procedure was performed in a limited *q*-range, namely, at *q* > 0.017 Å^−1^, to avoid the region with interparticle interactions. This value was used as *q*_*m*_ in the invariant calculation process. A Gaussian function was used to describe the particle size distribution and we have obtained a radius *R* = 159 ± 40 Å.

The cumulative delta invariant (Fig. 6B) behaves in accordance with the fit. Rapid growth is observed at a range up to *q*_*m*_ and stable behavior is observed at larger angles. Small fluctuations indicate an imperfect fit. The chosen *q*_*m*_ is labeled as a vertical blue line in Fig. 6B.

The data were on the absolute intensity scale, which results in a comparable value of *Δb*^*2*^ obtained using the proposed approach (1.04·10^20^ cm^2^/g^2^) to the theoretical value (1.197·10^20^ cm^2^/g^2^) calculated using Eq. (2).

The intensity *I(0)* was obtained by extrapolating the fitted curve to *q* = 0. At this point, we have all the components to use the classical equation for calculating the molecular weight. The obtained values of *M*_*w*_ within the concentration series we had, ranged from 5.0·10^7^ g/mol to 5.9·10^7^ g/mol.

### Poly(BuMA) latex (solid-state, membranes)

**Figure 7:**
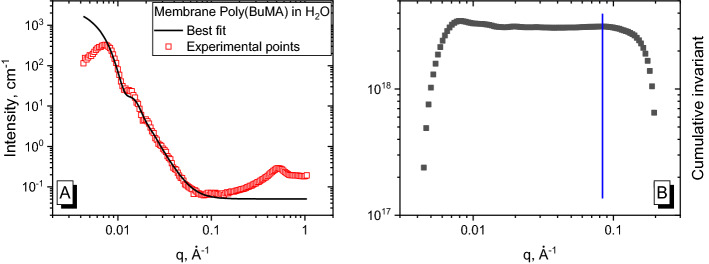
**(A)** Small-angle X-ray scattering curves of poly(BuMA) membranes in water with its best fit and **(B)** cumulative invariant with the labeled *q*_*max*_ value as a vertical blue line.

Another candidate for molecular weight estimation is poly(BuMA) with a relatively high concentration, namely, 0.477 wt.% in water. The scattering curve is shown in Fig. 7. The density of poly(BuMA) ranged from 1.05–1.06 g/cm^3^ and resulted in a wide range of theoretical values of *Δb*^*2*^ = 1.0328·10^19^ to 1.6520·10^19^ cm^2^/g^2^.

The scattering curve was fitted using the spherical function, with R = 346.7 Å. By applying the proposed method for estimating *M*_*w*_, we have obtained *Δb*^*2*^ = 1.35·10^19^ cm^2^/g^2^ and *M*_*w*_ = 1.38·10^8^ g/mol. Another form factor that describes the scattering curve better in terms of χ^2^ is the ellipsoid function with a polar radius of 259 Å and equatorial radius of 433 Å. In this case, we obtained *Δb*^*2*^ = 1.27·10^19^ cm^2^/g^2^ and *M*_*w*_ = 1.27·10^8^ g/mol. With expected value of *M*_*w*_ = 1.28·10^8^ g/mol, both approaches produce correct estimation of *Δb*^*2*^ and *M*_*w*_ values, while the ellipsoid model is expectedly much closer to the truth.

It is important to note here the choice of the *q*_*max*_ value. The fit of the whole accessible experimental range is used to assure the correct prediction of scattering curve behavior at low *q*-region. The cumulative invariant is used as a graphical measure of the fit quality. At higher *q*-values, the value of invariant decreases rapidly due to the non-fitted features in that region. It means that the chosen model is incomplete and does not describe features at a lower scale. As far as those features do not influence the behavior of the scattering curve at the lower *q*-region, one should obtain the correct estimation of molecular weight. In other words, if the proposed model is correct, one can choose *q*_*max*_ in range 0.015 Å^-1^ < *q* < 0.085 Å^−1^. The calculated *M*_*w*_ would be the same in all cases.

### Apoferritin

The proposed method is very useful for determining the molecular weight of proteins. The approach would avoid the use of a series of concentrations, as the proteins might be very expensive. Moreover, Russel^13^ has revealed some problems that may occur with an absolute intensity scale determination.

**Figure 8:**
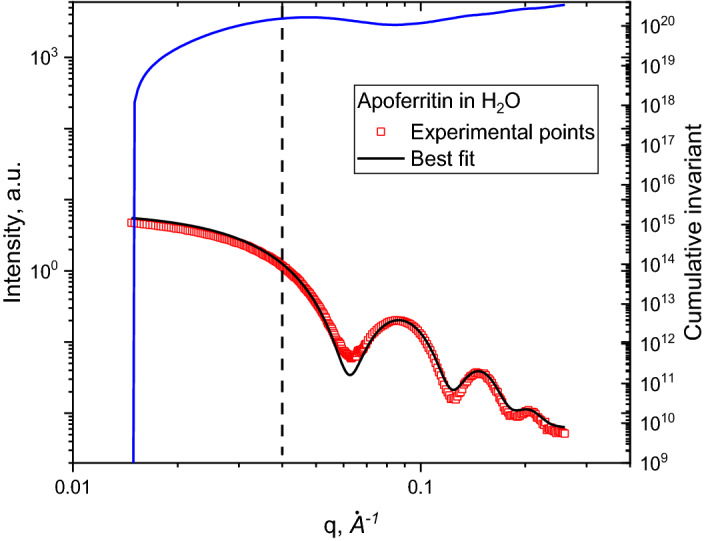
Small-angle X-ray scattering curves of apoferritin. Red squares correspond to experimental points, the black line corresponds to the best fit and the blue line corresponds to the cumulative invariant.

We decided to estimate the molecular weight of apoferritin, the iron-free ferritin protein, to choose a slightly more complex particle shape. The appropriate scattering curve is presented in Fig. 8. E. Mylonas and D. Svergun used this protein along with many other tested proteins to determine its molecular weight^2^. In their approach, apoferritin was an outlier, exhibiting sometimes up to a 20% deviation.

Apoferritin with a concentration of *c* = 47.2 mg/mL was dissolved in 0.135 M NaCl. We performed all the same steps, starting with the fitting procedure. The shape of this protein is well-known. The “Spherical Shell iii” model from SASFit package was used. We excluded the *q*-range that might be affected by particle interactions (*q* > 0.4) and subsequently extrapolated the model with the obtained parameters to the entire available range. The obtained curve is presented in Fig. 8 as a solid black line. The choice of *q*_*max*_ is rather straightforward. With a perfect fit, we would expect no difference between the experimental and theoretical curves at *q* > 0.4. Thus, it should exert a close-to-zero effect on the cumulative invariant at larger angles. With a perfect fit, we can choose any value of *q* that is larger than 0.4. In our case, the fit is not perfect, and thus we stopped on exactly that value to reduce effect of the imperfection.

Apoferritin is sometimes used as a standard for molecular weight determinations. The value of *M*_*w*_ obtained using our method is 418 000 g/mol, which is very similar to the well-known molecular weight of apoferritin, which is 440 000 g/mol. This example perfectly shows the case where the correct prediction of the scattering curve at low q-range leads to the correct *M*_*w*_ estimation even though the fit is not perfect in higher q-range.

If the model is not known a priory, Guinier approximation could be used to predict the scattering curve behavior at low-*q* region. In this case, higher accuracy could be obtained only through additional measurements of different concentrations to get the correct *q*_*max*_ value as it is shown in Fig. 1A.

### Lysozyme C and mycobacterial suicide toxin

For the case of proteins, where lower concentrations are usually used, the approach described in “Case of a low concentration” is more appropriate.

The experimental data were taken from SASBDB database^14^.

For the Lysozyme C, entry SASDAC2 was used, where the important low-*q* range is covered. The expected molecular weight, calculated from the deposited sequence and ligand information for Lysozyme C is 14 kDa; the concentration, used in the experiment, is 8.09 mg/ml. Average partial specific volume for proteins is usually taken as 0.708 mL/g. For the case of low concentration, we do not need to build a full model, and Guinier approximation would be enough to determine intensity, extrapolated to *q* = 0. The Guinier approximation gave us *I*_*0*_ = 12.08 and *R*_*g*_ = 15.5 Å, which is close to the value reported in entry SASDAC2. At this point, we have everything we need to estimate the molecular weight using Eq. (14). It does not matter if the scattering curve is on an absolute scale or not: scaling would result in the same coefficient in both the numerator and denominator.

$${M}_{w}=\frac{2{\pi }^{2}{I}_{0}{N}_{a}\left(\frac{1}{\stackrel{-}{V}}-c\right)}{\underset{0}{\overset{\infty }{\int }}I{q}^{2}dq}=14557 g/mol$$

The calculated value, 14.5 kDa, is very close to the expected molecular weight, 14 kDa.

Similar accuracy was obtained for Mycobacterial suicide toxin (entry SASDD43). The obtained *M*_*w*_ value is 19 kDa with expected 20 kDa.

This method is more sensitive to the signal-to-noise ratio as it is using the whole scattering curve. The accuracy could be improved by the smoothing of higher-*q* range.

## Conclusions

The validity of the proposed method was confirmed by comparing the obtained *M*_*w*_ value with the value calculated using two other methods:

- *M*_*w*_*(V)*: For a dry (non-swollen) particle, *M*_*w*_ could be calculated from the volume
- *M*_*w*_*(I)*: Classic method based on absolute intensities.

*M*_*w*_*(V)* and *M*_*w*_*(I)* should be equal, as they are given by the same distribution moment.

### Advantages of the method

- One of the best benefits of this method is that it is allows the molecular weight to be calculated on a relative scale using only one scattering curve.
- The method allows the calculation of molecular weights for concentrated solutions.
- The proposed method allows measurements of particles dispersed in a solid material (matrix). Particles in the solid-state do not affect the reliability of the results.
- The proposed method does not depend on particle swelling, and moreover, it facilitates the calculation of the swelling ratio.
- Based on the behavior of cumulative invariant, one can judge the validity of chosen model for fitting the scattering curve.
- The proposed method allows *M*_*w*_ to be determined without using *q*-regions, which may not be available (very small *q*) or if the values in those regions are not trustworthy (large *q*).

### Limitations of the method

- Obviously, due the interaction-based character, this method will fail for:
  - highly-charged particles
  - anisotropic particles
  - multi-component particles
- Every parameter that affects the low *q*-range, including the presence of aggregates or very wide polydispersity, is in conflict with expected effect of the concentration change and thus should be avoided.
